# Involvement of vasoactive substances in hemodynamics disturbances in cirrhosis

**Published:** 2014

**Authors:** M Grancea-Iancu

**Affiliations:** * Department of Internal Medicine and Rheumatology, “Dr. I. Cantacuzino” Hospital; “Carol Davila” University of Medicine and Pharmacy, Bucharest, Romania

**Keywords:** Cirrhosis, hepatic stellate cell, vasoactive substances, oxidative stress

## Abstract

Cirrhosis is a pathological entity characterized by the association of hepatocyte necrosis, fibrosis and regenerative nodules; hemodynamic and neurohormonal metabolic factors intervening in its development mechanisms, resulting in hepatic stellate cell activation and transformation and development of liver fibrosis.

Cytokines are key modulators of liver cell fibroblast transformation. Prostaglandins play an important role in the control of vascular tone and in thrombosis; Angiotensin II stimulates fibroblast proliferation by AT-1 receptors. Thrombin influences cellular remodeling in the liver and cardiovascular cirrhotic patients. Oxidative stress is involved in the development of liver cirrhosis by primary and secondary biological irreversible effects. Complex etiology involving vasoactive substances, oxidative stress in the pathogenesis of liver cirrhosis, require further studies to elucidate the mechanisms involved in hemodynamic disturbances associated with this disorder.

## Introduction

Cirrhosis is a pathological entity characterized by the association of hepatocyte necrosis, excessive fibrosis and regenerative nodules, leading to an irreversible alteration of liver architecture with significant hemodynamic and biological effects. The development and evolution mechanisms involved cirrhosis hemodynamic and neurohormonal metabolic factors resulting in hepatic stellate cell (HSC) activation and transformation under the influence of cytokines, the production of collagen type I, III and IV and the development of liver fibrosis. 

**Fig. 1:**
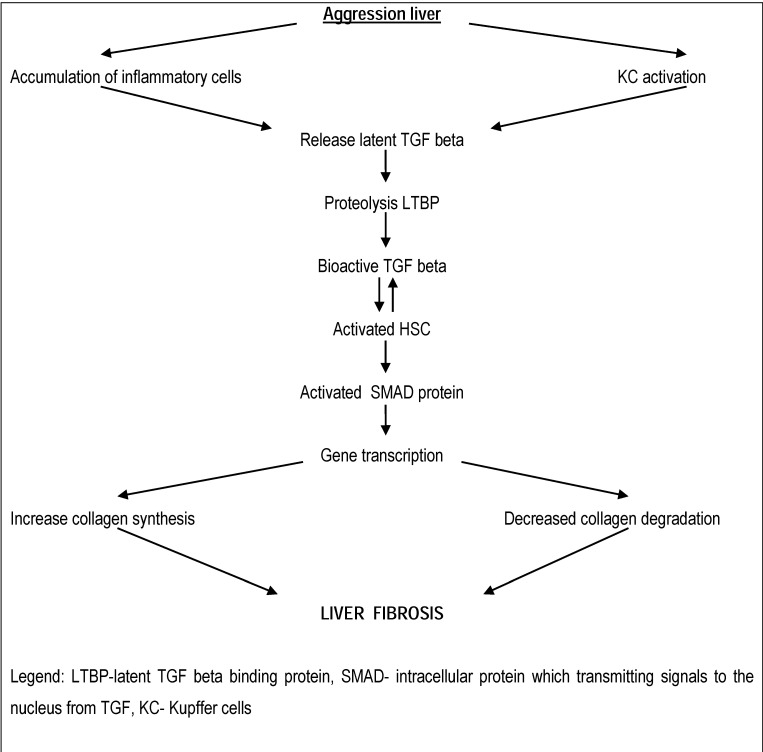
TGF morphopathogenesis

Cytokines are key modulators of stellate cell fibroblast transformation. TGF-beta1 transforms the growth factor beta mRNA which is present in large amounts in the liver, stimulating fibrogenesis. Characteristics include the presence of TGF pathogenesis of inflammatory cell infiltrate and localized in the liver parenchyma (**Fig. 1**). 

Recent studies demonstrate that the use of low molecular weight antigenic peptide autoantibodies cause a substantial reduction in TGF-beta1 and liver fibrosis [**1**]. Proinflammatory TNF-alpha activates HSCs Mechanisms and stimulates the synthesis of fatty acids causing fatty liver uploading. The increase in gene expression of neutrophils and production BPI (protein cell growth) in cirrhotic patients is directly proportional to the levels of TNF-alpha. It has been shown that cirrhotic patients Child C class BPI production is significantly increased compared to patients classified as Child B, perhaps because it plays a regulatory role in antagonizing proinflammatory mechanisms mediated by TNF-alpha [**2**]. IL1 and IL6 lymphocyte cytokines are directly involved in stimulating hepatic stellate cell. IL6 may increase mRNA transcription rate and IL1 has a major role in hepatic fibrogenesis in vivo by stimulating the production of 3D Coll [**3**].

Prostaglandins (PGE) play an important role in controlling the vascular tone and the process of thrombosis, relaxing smooth muscles. PGE2 causes vasodilation, derived from COX-2 (PG synthase enzyme that catalyses and it is involved in hepatic stellate cell functions) and inhibits platelet growth factors resulting in the proliferation and the transformation of TNF-beta 1. Chronic Epoprostenol therapy appears to be a promising treatment of porto-pulmonary hypertension.

**Renin-angiotensin-aldosteron system**

Renin activates the conversion of angiotensinogen in Ang I (angiotensin I), which is turn to Ang II (angiotensin II) and has an overwhelming importance like the growth hormone in liver fibrosis. Ang II acts through AT-1 receptors and MAP kinase and modulates hepatic hypertrophic response by inducing genes for transforming factor TGF-beta1. Thus, fibroblast proliferation is stimulated. Recent studies have shown that AT-1 receptor blockade prevents Ang II effects on hepatic stellate cells and reduces the degree of liver fibrosis [**4**]. Losartan has been shown to reduce short-run fibrosis by setting the AT-1 receptor [**5**]. Recent studies have shown that the AT-1 receptor blockade prevents Ang II effects on hepatic stellate cells and reduces the degree of liver fibrosis [**4**]. Losartan has been shown to reduce short-run fibrosis by setting the AT-1 receptor [**5**]. 

**Endothelin and nitric oxide**

Vascular endothelium, probably the largest endocrine organ, plays a major role in the hemodynamic disturbances in cirrhosis. EDRF (endothelial-derived releasing factor) is one of the most important vasodilators, its disruption from various reasons leading to an impaired blood flow, development and evolution of thrombosis and inflammation. Endothelial synthesized from the L-arginine amino acid has been identified as nitric oxide (NO), by the action of the NO-synthase. A decrease in the activity or expression of eNOS (endothelial NO synthase) in patients with cirrhosis, leads to the NO depletion which causes vasoconstriction, decreased blood flow and accelerates fibrosis. Portuguese Studies revealed altered plasma levels of intracellular secondary messengers NO (cGMP) in patients with cirrhosis [**6**]. Plasma levels of NO and cGMP are significantly higher in patients with decompensated cirrhosis versus compensated and compensated cirrhosis versus healthy individuals [**7**]. The endothelium is an important source of vasoconstrictor factors, the most well known being the endothelins. Endothelin 1 (ET-1) is a 21 amino acid peptide, synthesized interendothelial, which causes prolonged and pronounced vasoconstriction by regulating vascular tone and is involved in the development of portal hypertension and porto-pulmonary syndromes. Two endothelin receptors, ET-A and ET-B, have been described. This receptor and the enzyme inhibitors may be useful in counteracting endotelio convertor effects associated with abnormal vascular vasoconstriction. Bosentan, an antagonist of ET-1 is used in porto-pulmonary hypertension of liver transplant patients.

**Thrombin**

Anglo-American studies have shown that thrombin stimulates the production and secretion of extracellular proteins and cellular remodeling at liver and cardiovascular system in patients with liver cirrhosis [**8**]. Most of these effects are mediated by PAR (protease-activated receptor) which is stimulated by thrombin and is expressed by platelets, endothelial cells and hepatic stellate cells.

**Oxidative stress (OS) and oxygen free radicals (OFR)**

**Fig. 2:**
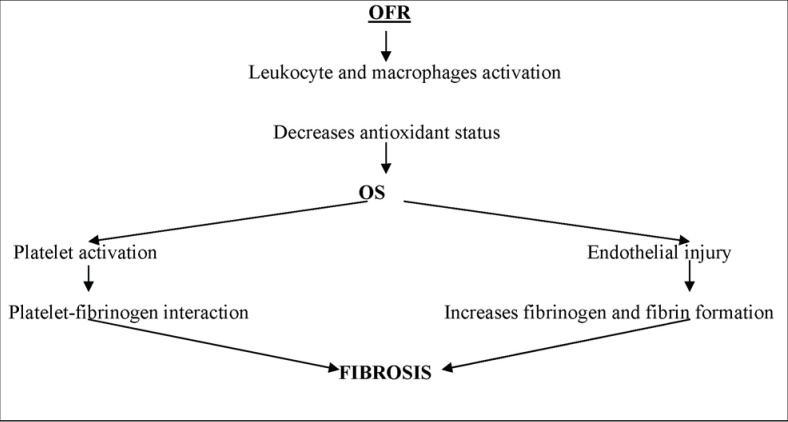
The role of oxygen free radicals (OFR) in the cell’s fibrosis

Oxidative stress results from an imbalance between the formation of oxygen free radicals (oxidants) and the amount of antioxidants in favor of the first. Oxidative stress is reached as a result of massive non-synthesis of oxygen free radicals (RLO) or the alteration of antioxidant mechanisms. Recent studies demonstrated the involvement of OS in the development of liver cirrhosis by primary and secondary irreversible biological effects (**Table 1**) [**9**]. In liver cirrhosis, the presence of OS and inflammation are directly related to the endothelial dysfunction present in this condition (**Fig. 2**). A longer period of stress means that the negative effects of OS occur due to the depletion of antioxidant capacity. It has been shown that the administration of L-arginine through its antioxidant effect lowers OS and reduces the development of liver fibrosis. The inflammation process is the most important free radical generator. Types of the reactive oxygen species are molecular oxygen, superoxide, hydrogen peroxide, hydroxyl radical, nitric oxide. The OFR role is essential in producing inflammation and apoptosis, necrosis and cell dysfunction. In this process, a series of chemical mediators is activated. It has been shown that the production of reactive oxygen species depend on the nature of the phagocytosed cell [**10**].

**Table 1:** Liver biological consequences of oxidative stress

	LIVER BIOLOGICAL CONSEQUENCES OF OXIDATIVE STRESS
Primary reversible effect	- decreased energy production
	- fatty acid peroxidation of cell membranes
	- modification of unor cellular metabolic processes due to alteration of protein
	- decreases of antioxidants
	- changes in the cell membrane, ion imbalance
Secondary irreversible effect	- calcium ion imbalance
	- cell lysis
	- disorders of blood circulation
	- the nucleic acid and protein peroxidation
	- functional disorders and local damage
	- decreased body resistance

## Conclusions

Hepatic cirrhosis is characterized by a complex pathogenesis of various factors involved in vasoactive action, which causes hemodynamic associated abnormalities.
